# From Enchantment to Disillusion: A Narrative Exploration of Cannabis Use Disorder Among Young Israeli Combat Veterans

**DOI:** 10.3389/fpsyt.2021.643618

**Published:** 2021-06-18

**Authors:** Miri Serebro, Shira Sobol-Goldberg, Daniel Feingold

**Affiliations:** ^1^MA Program, Psychology Department, Ariel University, Ariel, Israel; ^2^School of Social Work, Bar-Ilan University, Ramat Gan, Israel; ^3^Psychology Department, Ariel University, Ariel, Israel

**Keywords:** cannabis, disorder, Israeli, veterans, disillusion, trauma

## Abstract

**Introduction:** Substance use is common among military personnel and war veterans, especially combat veterans. Despite substantially high prevalence of cannabis use and Cannabis Use Disorder (CUD) consistently reported among veterans, little is known about psychological factors which may underlie CUD among this population.

**Methods:** In this study, we used narrative analysis in order to interpret retrospective in-depth interviews of combat veterans (*N* = 12) who were released from mandatory military duty during the past 5 years and currently qualified for a diagnosis of CUD. Participants were recruited from a larger quantitative study were eligible for participation if they screened positive for a diagnosis of CUD according to the Cannabis Use Disorder Identification Test- Revised (CUDIT-R) questionnaire. CUD diagnosis was validated in-person using the cannabis section of the Alcohol Use Disorder and Associated Disabilities Interview Schedule-5 (AUDADIS-5) interview protocol. All interviews were transcribed and coded using the content analysis procedure.

**Findings:** Five main themes were extracted: (a) Traumatic events (b) Attitudes toward cannabis use (c) Combatant identity (d) The role of authority/father figures, and (e) Moral crisis. A meta-theme has been identified, “from enchantment to disillusion,” representing a gradual psychological shift from a hopeful, highly motivated stance into the current state of mental rupture and moral injury, which are unsuccessfully compensated by excessive use of cannabis.

**Conclusions:** This study shed light on the etiology of CUD among young combat veterans, highlighting the role of supposed self-medication for trauma and sense of betrayal.

## Introduction

Cannabis is the most commonly used drug, globally, with an estimated 192 million people, equivalent to 3.9% of the world population, who used cannabis during 2019 (1). In the past decades accumulating evidence has emerged associating cannabis use with several adverse long-term effects, particularly among individuals who use high-potency cannabis and those who use it frequently (2). One such risk is developing Cannabis Use Disorder (CUD), a clinical condition associated with cannabis use which lasts at least 12 months, characterized by physical and psychological dependence as well as functional impairment caused directly by the use of cannabis. A 2.5% past-year prevalence of a CUD diagnosis according to the Diagnostic and Statistical Manual of Mental Disorders, 5th Edition [DSM-5; (3)] has been reported in the U.S (4), with ~27% of lifetime cannabis users developing CUD (5).

Several factors have been associated with increased risk for transition to CUD. For example, higher frequency and quantity of cannabis use (6), co-occurring psychiatric disorders, early initiation of cannabis use and childhood traumatic events (5). Military veterans have been identified as a population highly inclined toward cannabis use and CUD. Following nicotine and alcohol, cannabis is the most commonly used addictive substance among veterans (7), who commonly initiate cannabis use following their release from duty due to its legal status (8). Approximately 70% of veterans who initiate cannabis use will become regular users, and more than 20% will develop a DSM-IV diagnosis of cannabis abuse or dependence (9). It has been reported that between 2002 and 2009, prevalence of cannabis dependence has nearly doubled among U.S. veterans (10).

It has been suggested that combat veterans are specifically more prone to cannabis use and CUD compared to non-combat veterans (11). This may be attributed to various factors, including high rates of chronic pain among combat veterans (12). Furthermore, combat exposure has been associated with increased risk for frequent cannabis use (13), presumably due to the mediating role of post-traumatic stress symptoms and Post-traumatic Stress Disorder (PTSD), which has been extensively studied as a risk factor for developing CUD among veterans. Notably, as other factor may also underlie combat veterans' proneness to CUD, the use additional research methodologies has been recently encouraged (12). For example, growing attention is drawn to the effect of moral injury, a shame and guilt-based trauma-related syndrome which may develop among veterans, and its effect on various future outcomes (14, 15). However, yet little is known concerning its contribution to CUD (13), nevertheless in qualitative studies.

‘To the best of our knowledge, little is known concerning combat veterans' narratives regarding cannabis use and CUD. In a recent study, Krediet et al. (16) interviewed a focus group comprised of Dutch veterans for whom cannabis was prescribed for medical purposes. Participants emphasized using cannabis for the purpose of attenuating their post-traumatic symptoms, primarily sleep disturbances, and were reluctant to report using cannabis for additional purposes. However, this study focused on medical marijuana users and could not be generalized to recreational cannabis use and CUD (17).

In Israel, rates of cannabis use within the general population were traditionally lower compared to the U.S. and Europe (18). However, by 2016, rates of cannabis use have increased substantially from ~9 to 27% for past-year prevalence (19). In a recent investigation among Israeli combat veterans, more than 50% reported using cannabis in the past year (13). Military service in Israel is mandatory, lasting for 30 or 36 months for women and men, respectively. Israeli combatants have historically enrolled in two primary duties. The first is conventional warfare surrounding the country's borders, including traditional combat-related experiences such as being attacked or ambushed (20). The second includes policing and confrontation with the Palestinian civilian population in heavily-populated urban environments (21).

In this study, we sought to explore narratives of Israeli combat veterans recently released from military duty, and who currently qualified for a CUD diagnosis. In particular, we aimed to explore the extent to which major life events prior to, during and following military service emerge as correlates of transition to CUD. An a-priori hypothesis (22) was that lifelong traumatic events, including life-threatening events associated with PTSD (12) as well as morally conflicting events which are common in battle (13), will emerge as themes among combat veterans with CUD.

## Methods

### Participants

Participants were 14 individuals who were recruited from a larger quantitative study focusing on veterans recently released from combat duty who had used cannabis regularly (at least 3 days per week) during the past 6 months. Characteristics of study participants are presented in Table 1. All names were amended and personal data disguised in order to maintain participants' anonymity. Participants were recruited to the quantitative study *via* social networks (Facebook, WhatsApp, etc.) and were included in the study if they served in a combat unit for at least 1 year (out of the three mandatory for men in Israel). Participants were excluded if they reported being prescribed medical marijuana during the past 6 months, due to the lack of validated measures assessing CUD in the medical context (17). Upon completion of the quantitative study, participants were asked if they were willing to participate in a face-to-face interview in case they qualify for the inclusion criteria of the qualitative phase, i.e., screening positive for a DSM-5 diagnosis of CUD.

**Table 1:** Personal characteristics of study participants (*N* = 12).

**Name**	**Age^**a**^**	**Military service duration**^**a**^	**Military unit**	**Current status**
David	22	3	Armored Corps	Musician
Jonathan	25	3	Infantry	Student in the social sciences
Guy	25	3	Infantry	Student in the social sciences
Tom	27	7	Special forces	Student in the social sciences
Eyal	25	3	Infantry	Teacher
Sharon	25	3	Combat intelligence	Engineering student
Avishai	27	3	Combat engineering	Student in the exact sciences
Alexander	26	3	Special Forces	Engineering student
Eilon	26	3	Armored Corps	Student in the human sciences
Michael	26	3	Infantry	Engineering student
Eden	31	8	Infantry	Student in engineering (MA level)
Noam	23	3	Armored Corps	Student in the social sciences

a *In years*.

### Measures

#### Cannabis Use Disorder Identification Test Revised (CUDIT-R)

This instrument was used in the quantitative phase in order to screen for participants who qualified for a DSM-5 diagnosis of CUD, and were thus eligible for participation in the qualitative phase. The CUDIT-R is an eight-item self-report measure assessing problematic cannabis use during the past 6 months (e.g., “How often during the past 6 months did you need to use cannabis in the morning to get yourself going after a heavy session?”). Items were rated on a five-point Likert scale ranging between “0” (never) to “4.” The sum score was automatically computed and a cut-off point of 6 was used to initially screen for a DSM-5 diagnosis of CUD (24). The CUDIT-R has previously shown good reliability (α = 0.914 and α = 0.83) in two separate samples (23, 24).

#### Alcohol Use Disorder and Associated Disabilities Interview Schedule-5 (AUDADIS-5)

A structured face-to-face diagnostic interview developed by the NIAAA and designed for lay interviewers. Among other issues, the AUDADIS-5 addresses DSM-5 substance use disorders. For the current study, the cannabis section of the AUDADIS-5 was used (25), which has previously shown good test-retest reliability for a past-year diagnosis of DSM-5 CUD (kappa ≥ 0.60) in a general population sample (26).

#### In-Depth Interviews

Interviews followed a timeline procedure (27), focusing on two timelines: (a) major positive and negative life events (b) trajectories of cannabis use, including initiation and cessation of cannabis use, increase/decrease in frequency of cannabis use and alleged onset of CUD (27). In order to allow participants to freely express their narratives, no topic list was used (28). However, interviewers emphasized three lifetime periods: (1) prior to military service, including early trauma, substance use during adolescence and expectancies toward military service (2) during military service, including training period and combat deployment, and (3) following release from duty, including adjustment to civilian life and major developmental stages during emerging adulthood.

### Procedure and Analyses

Participants in the quantitative study, who qualified for a probable diagnosis of CUD (CUDIT-R ≥6) and who gave their consent for participation in the quantitative study, were approached *via* phone by one of the researchers or a research assistant. Time and place for the face-to-face interviews were set, and participants were requested to attend the interview while not under cannabis intoxication. Interviews were held primarily at the authors' offices, with duration ranging between 60 and 90 min. Prior to initiation of the interview, the AUDADIS-5 was administered in order to validate a probable diagnosis of DSM-5 CUD (i.e., meeting at least two CUD criteria). Two participants did not meet CUD diagnostic criteria according to the AUDADIS-5, and were therefore not included in the data analysis. Upon completion of the interview, participants were given 150 NIS ($\tilde{=}$45 U.S Dollars).

All eligible interviews were audio recorded and transcribed while omitting personal details which could allow identification. Transcribed interviews were analyzed using the content analysis procedure (29), initially coding semantic segments and subsequently extracting a-priori and post-priori themes and meta-themes emerging from the interviews (22). In order to increase inter-rater reliability, initial coding was conducted by two independent raters (DF, MS) (30). Coding and theme extraction were conducted using the ATLAS.it software for qualitative data analysis. The study was approved by the Institutional Review Board (IRB) at Ariel University.

## Results

**Figure 1:**
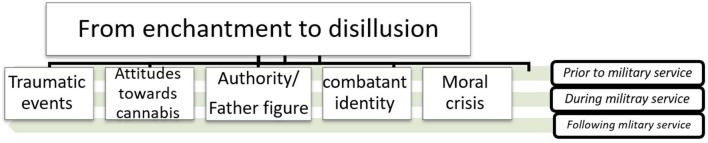
Extracted themes and meta-theme.

Data analysis has yielded the following themes, which will then be presented in detail below: (a) Traumatic events (b) Attitudes toward cannabis use (c) Combatant identity (d) The role of authority/father figures, and (e) Moral crisis (Figure 1). In line with our preliminary hypothesis, these themes followed a timeline, representing three major periods: prior to, during and following military service. Table 2 describes frequency of the primary themes which emerged from the qualitative analysis. A meta-theme that has been identified is “from enchantment to disillusion.” This meta-theme represents a narrative shift which is reflected in the extracted themes. This narrative shift represents a gradual psychological and behavioral transition from an “enchanted” stance, in which participants embraced and identified with values and norms associated with cannabis use, as well as personal and national military ethos. Eventually, participants' narratives reflected a sense of “disillusionment” from this identification, accompanied by an experience of mental and moral disenchantment.

**Table 2:** Frequency of themes emerging from the qualitative analysis.

**Theme**		**# Of participants who mentioned the theme**
Traumatic events	During military service	6
	During operation protective edge	6
Cannabis: attitudes	Coping *via* cannabis use	10
	Ambivalent Stance	11
Authority	The role of the commander as a parental figure	4
	The role of the commander: disillusionment	10
Combat identity	High expectation toward military service	8
	Disappointment with military service	9
Moral crisis	Moral injury	5
	Sense of betrayal	11

### Traumatic Events

A main, a-priori theme that emerged had to do with traumatic events and their effect on patterns of cannabis use. The majority of participants in our study were drafted into their service during Operation Protective Edge, which took place in Gaza strip in 2014, and was characterized by a relatively high number of IDF casualties. Its effects were repeatedly reflected in participants' narratives. For example, Tom recalled:

“*Only a minute ago we hung out together, he was my friend… and then a moment later he's dead. And there was this guy who went to high school with me, and he's missing in action. And these three guys from my unit, great fellows, we used to spend a lot of time together, they're also killed. It's a deadly period, the worst that could possibly be, it's the first time that people I know go away and never return. It's a time when I understood that death is eternal.”*

### Attitudes Toward Cannabis Use

All participants related to cannabis as a means for coping with life's adversities and traumatic events from their military service. The word “disconnection” repeatedly appeared when addressing the effects of cannabis use, echoing cannabis' role as a dissociative defense mechanism which allows participants to maintain such disconnection from traumatic memories and avoid mental pain.

“*Cannabis makes me calm. I love this sort of disconnection. It allows me to sit down at the end of the day and say: ‘I'm switched-off, I've forgotten everything that happened today.' If I don't smoke [cannabis] my thoughts run wild. When I smoke I just turn it off.” (Jonathan)…“Why do I smoke? Because I want to take some time off from my life. To get away and lay down on a cloud of laughter, forget all my worries and hardships” (Sharon)*

Some of the participants specifically addressed cannabis use as a means for medicating trauma-related experiences. The most common was sleep disturbances, while others addressed the compensatory effect of cannabis:

“*For a long time I could not sleep. It was hard for me and I tried all sorts of solutions. Cannabis made it better. It's not only more effective than other medications, but it also really works… I can finally sleep properly. For time to time there are tough nights, but it became a part of my life in a way that I don't think I'll ever want to quit” (Eyal)…“[Cannabis] allows me to cope with the losses I have suffered”* (David)…“*I told myself: I made it through this war [Operation Protective Edge], nothing happened to me. I didn't die, I'm not wounded, I deserve to do whatever I want”* (Guy)

As the interviews progressed, participants expressed an ambivalent stance toward cannabis. On the one hand, they addressed the regulatory function as a means for comprehending traumatic memories without being emotionally shaken. On the other hand, participants mentioned tolerance, psychological dependence and loss of control as common phenomena associated with cannabis use, and at times even fear of its negative long-term effects. Participants also mentioned that in some cases cannabis may increase their experience of negative emotions, such as anxiety, post-traumatic stress symptoms and anhedonia. In addition, a feeling that was portrayed is one of false wellness which emerges after prolonged cannabis use.

“*I'm starting to feel bad about the weed. When you're weak you start to see its downsides. If we would have talked six months ago, I would say it's the best.” (Michael)…“In a way I blame the cannabis. It gave me more anxiety, more worries, more demons, many things that I never had before. It brings me down a little. I know I blame it all on a plant, but I really think it has a big part in this”.“It makes you feel good in a false way, because at some point your body gets adjusted to the feeling. I feel I want to try and quit, I feel it turns my motivation down, the will, the passion. I don't like it when that happens. It turns my life off.” (Avishai)*

### Combatant Identity

Participants reflected on the high expectations that preceded their recruitment to combat units. Some emphasized the fulfillment of a national ethos, while for some becoming a combatant followed a family heritage and destination:

“*At the beginning it was so exciting. My heritage. What my father kept talking about his entire life. His comrades and the strong bonds they have, this is something I wanted for myself.” (Jonathan)…“Everyone would praise us, telling us how important we are to the country, telling us we're heroes, and all sorts of other clichés” (Sharon)*.

However, the majority of participants experienced a deep sense of disappointment following their military service, accompanied by feelings of distrust. Some participants talked about the discrepancy between their anticipation for an exciting and meaningful service, amplified by a national glorification of the Israeli combatant myth, and facing the monotonous, sometimes boring, military routine. Even while taking part in combat missions, a gap emerged between high expectations for flawless warfare and the disorganized, at times chaotic conduct of their combat unit:

“*it gets tough when you realize you're not going to fight on a daily basis. I wanted to do more, but this routine of walking around doing nothing kind of brought me down” (Jonathan)…“So many mistakes have been made when it came to people's lives. I have a friend who died from a mortar bomb while sitting in a rally point. He only had two seconds to seek cover in some concrete cylinder that no one can really squeeze himself into. This just isn't right” (Eyal)*.

### Role of Authority/Father Figures

It appears as though a part of the anticipation toward military combat service had to do with the image of command. Several participants related to the commander as a parental figure whose responsibility over his subordinates was absolute. Notably, this followed participants' personal biography, as some grew up with a dominant father figure, while others reported being deprived of a close relationship with a father figure:

“*He's [the commander] in charge of 20 soldiers. It's a matter of life and death, so he has to be their father. He's in charge of everything and if he's dysfunctional, he has to have a good reason for that” (Tom). “I wanted to be posted exactly where my father was, go to the same places as he did, as a paratrooper” (Jonathan*).

These expectations toward their commander were often faced with a disillusioning reality in which commanders failed to be flawless, and at times were perceived as unfair, irresponsible and ego-driven:

“*There are several troop leaders who have lost their humanity and trampled me along the way. Let's just say that during Operation Protective Edge, the most negative psychological experiences I had are related to the way I was treated by commanders, not to what I have seen in action”(Guy)… “The commander yelled ‘casualties!', so being a paramedic I took my stretcher and ran like crazy to see who's injured. I was certain there's someone dying out there, but it turned out to be a drill, a way to see how we respond. What a cruel trick to pull” (David)*.

### Moral Crisis

During the interviews, participants often recalled morally injurious events from their military service. These events seem to create a prolonged tormenting conflict between participants' internalized moral values and the immoral deeds they were bound to perform as soldiers. In some cases, immoral conduct emerged from the paradoxical nature of the battlefield, while in other cases participants were ordered to perform immoral acts. At times, moral injury has occurred while in action, and in some cases it emerged later on after their release from duty. For many of the participants, these “faulty events” remain an unsettled business, accompanied with shame and guilt, which still echo in their daily life, self-perception and their desire to self-medicate:

“*I felt like I lost my identity while on duty. I used to look at myself in mirrors of the houses we seized in Gaza, and I got sick of seeing my face camouflaged, hearing little girls cry. I was like a mission contractor during these years, and it left its mark, I've lost my innocence back there” (Tom)… “90% of the time using such brutality was uncalled for. Punching someone just to keep him silent. For six months I would go out at night, see some farmer and chase him down with two assault dogs, only because he was allegedly filming and spying on us. But I don't believe it. This is what they [commanders] would tell me to do and I had to do it, but it felt faulty to me most of the time” (Avishai)…”*

## Discussion

In this study we explored narratives of Israeli veterans who were recently released from military duty and are currently qualified for a diagnosis of CUD. Major themes that emerged were related to trauma and moral crisis associated primarily with military-related events, as well as an ambivalent stance toward cannabis use, combatant identity and authority/father figures. A meta-theme that has emerged, encompassing these themes, is participants' gradual transition from enchantment to disillusion, resulting in a current cannabis-related pathology accompanied by an ambivalent representation of their military service.

In our study, trauma has emerged as a primary theme, and one that is highly concurrent with cannabis use. Previous findings have indicated that post-traumatic stress response and PTSD are very common among combat veterans and are highly comorbid with cannabis use and CUD (12). High comorbidity is also present within the general population, with epidemiological findings associating childhood adverse events and PTSD incidence with higher odds for CUD onset (5). A key motivation for cannabis use presented by participants in our study was regulating or coping with negative emotions associated with traumatic events and moral injury. This is in line with numerous reports indicating that relief from negative emotional experience is a primary motivation for cannabis use (31–33). The short-term effects of cannabis use often include psycho-physical stress reduction and reinforcing psychoactive effects which allow for a shift in attention away from the aversive emotional state (34). Therefore, it is understood why ex-combatants, often haunted by their traumatic past and conflicted present, may wish for the temporary comfort in cannabis use.

Despite its beneficial short-term effects on negative emotions, coping-oriented motives for cannabis use may in fact be harmful in the long-run. For example, coping motives for cannabis use have been associated with an increased risk for CUD onset (35, 36). It is often suggested that individuals may turn to substance use in order to compensate for their impaired or insufficient innate self-regulatory mechanism (33), eventually developing pathological patterns of substance use. While reward-driven motivation has been historically associated with the neuro-etiology of substance use disorders, it is now accepted that negative emotional states play a key role in the transition from substance use to SUD (37). By triggering negative rather than positive reinforcement, negative emotions may trigger compulsive or chronic use of substances, including cannabis (38–40). Thus, it may well be that veterans who cope with traumatic events and moral injury “self-medicate” their distress by using cannabis (41), are eventually inclined to develop CUD due to chronic dysregulation in reward and regulatory functions (42, 43).

In addition to the risk of developing CUD, participants emphasized the paradoxical nature of cannabis use in medicating post-traumatic stress symptoms. Despite accumulating evidence of the beneficial effect of cannabis use on sleep disturbances associated with PTSD (16, 44), cannabis use appears to be ineffective in long-term reduction of the majority of PTSD symptoms (45). Among heavy users, cannabis use may even be associated with a poorer PTSD outcome, namely increase in intrusive symptoms severity (46). While being generally ineffective in treating PTSD symptoms severity, veterans who use cannabis may be exposed to additional long-term adverse effects, such as panic attacks, psychotic episodes, cognitive deficit, etc. (12, 47).

Additional themes that emerged had to do with participants' disillusionment from a personal and national ethos related to the military, as well as a moral crisis associated with combat-related events. Several authors have emphasized the role of a sense of betrayal in the formation of moral injury among combatants and ex-combatants. In these cases, a deep sense of distrust may develop toward commanders and/or leaders, who are perceived as betraying 'what is right' in high stakes situations (14). A sense of betrayal is thought to increase vulnerability to post-traumatic stress symptoms, and at times provoke anger, hostility and aggression (14, 15). In some cases, betrayal may lead to shame, guilt and forms of self-destructive behaviors, including suicidality and frequent substance use (13, 48).

From a psychodynamic perspective, trauma may emerge from significant others' failure to provide a psychologically adequate environment for the development of a coherent and cohesive sense of self-esteem (49). Substance use is often conceptualized as an effort to medicate for an unstable or deprived sense of self-esteem (50). Therefore, it may well be that the transition reported by participants in their self-perception, from “heroes” to “mission contractors,” as well as their perception of authority figures, shifting from “parents” to “inhumane,” may result in an existential crisis which is compensated by excessive cannabis use (51, 52).

This study has several limitations that should be considered. First, the qualitative nature of the study doesn't allow for exploring statistical and causal association between variables, as well as for the generalization of the findings beyond the study participants (53). Second, being a qualitative study, the present investigation is influenced substantially by the subjective experience of participants, investigators and their interaction (54). Third, the relatively small sample may have halted the emergence of additional themes related to cannabis use and its psycho-social correlates. In addition, the lack of a control group, comprised of age-and-gender matched non-cannabis users or non-combat veterans who use cannabis, undermines the specificity of our findings. Fourth, this investigation focused on newly released, exclusively male combatants of Jewish ethnicity. Therefore, findings could not be generalized to older combat veterans, as well as non-Jewish veterans (Christian, Bedouin, Druze, etc.) and female veterans. The latter constitute ~2% of current combatants in the IDF and a growing proportion in the U.S. military. Female combatants' unique combat-related experiences are drawing increased scientific attention (55, 56) and should be subject to future exploration in the context of cannabis use and CUD.

Despite these limitations, the increase in the global prevalence of cannabis use and CUD, both within the general population and among veterans, as well as the emerging changes in cannabis' legal status, call for integrative research into the psychosocial predictors of CUD. Further exploration of veterans' narratives regarding cannabis use and CUD is needed in order to trace the etiology of CUD within this population. Themes that emerged from our study can be used for further quantitative investigation of underlying risk factors for CUD. The role of personal and national disillusionment, moral injury and the role of authority in predicting CUD among combat veterans should be explored in future large-scale longitudinal studies, thus allowing for better prevention, assessment, and treatment for those who develop pathological patterns of cannabis use.

## Data Availability Statement

The raw data supporting the conclusions of this article will be made available by the authors, without undue reservation.

## Ethics Statement

The studies involving human participants were reviewed and approved by Dr. Ephraim Grossman, Department of Education, Ariel University, Ariel, Israel. The patients/participants provided their written informed consent to participate in this study.

## Author Contributions

MS and DF: acquisition of data, analysis and interpretation of data, drafting the article, and final approval of the version to be submitted. SS-G: conception and design, analysis and interpretation of data, revising the manuscript critically for important intellectual content, and final approval of the version to be submitted. All authors contributed to the article and approved the submitted version.

## Conflict of Interest

The authors declare that the research was conducted in the absence of any commercial or financial relationships that could be construed as a potential conflict of interest.
